# The first German evidence-based clinical guidelines on borderline personality disorder

**DOI:** 10.1192/j.eurpsy.2023.789

**Published:** 2023-07-19

**Authors:** J. Stoffers-Winterling, K. Lieb

**Affiliations:** University Medical Center of the Johannes Gutenberg University Mainz, Mainz, Germany

## Abstract

**Introduction:**

Though the evidence regarding the psychotherapeutic and drug treatment of borderline personality disorder (BPD) is rapidly accumulating, with more then 120 randomised controlled trial on the clinical treatment being availabe currently, no evidence-based guidelines existed as yet in Germany. This talk will present the first German evidence-based clinical guidelines for BPD.

**Objectives:**

In this talk the German evidence-based clinical guidelines for borderline personality disorder will be presented, and focuses and important recommendations highlighted.

**Methods:**

A diverse, multidisciplinary panel, including members with research and clinical expertise in the treatment and care of individuals with BPD as well as representatives of persons with lived experience and their relatives, has developed the first evidence-based German treatment guidelines for BPD. The Australian NHMRC and the British NICE guidelines were used as source guidelines and adapted, based on the findings of updated literature searches. All recommendations were consented in an independently moderated, formalised consensus process. Special attention was paid to the management of financial and non-financial conflicts of interest.

**Results:**

The guidelines support the early detection and treatment of BPD and recommend that the diagnosis be made in adolescents from the age of twelve years on. Disorder-specific psychotherapies, i.e. structured psychological therapies that are specifically designed for people with BPD, are recommended as the first-line treatment for BPD. If the primary focus is the treatment of self-harm, Dialectical Behaviour Therapy or Mentalisation-Based Treatment are recommended. Drug treatment should only be considered if in crisis psychological interventions do not suffice and should be withdrawn as early as possible. Besides, drug treatment may only have a role if required for the treatment of comorbid psychic disorders. The guidelines also recommend educating significant others of the person concerned about the disorder and including them in the development of crisis plans. It is also recommended to offer support to relatives, especially if they are children or adolescents. Also, the role of individuals with BPD who are parents is considered, and early support to foster parenting skills and attachment relationships is recommended.

**Conclusions:**

The first evidence-based German guidelines on BPD are now available and will help to ameliorate the health care of persons with a diagnosis of BPD. The publication of an English version is impending.

**Disclosure of Interest:**

None Declared

